# Lifetime non-relational traumatic experiences are associated with biological ageing

**DOI:** 10.1007/s11357-026-02110-4

**Published:** 2026-01-28

**Authors:** Thole H. Hoppen, Nexhmedin Morina, Monica Aas, Julian Mutz

**Affiliations:** 1 Institute of Psychology, University of Münster, Münster, Germany; 2 Department of Clinical Psychology and Psychotherapy, University of Göttingen, Göttingen, Germany; 3 Social, Genetic and Developmental Psychiatry Centre, Institute of Psychiatry, Psychology & Neuroscience, King’s College London, London, UK

**Keywords:** Trauma, Biological ageing, Frailty, Metabolomics, Telomeres, Grip strength

## Abstract

Exposure to non-relational trauma, such as serious accidents, war or life-threatening illness, is linked to poor mental and physical health. Its relationship with biological ageing markers, however, remains underexplored. This study's aim was to examine associations between non-relational trauma and multiple biological ageing markers, and to assess whether associations vary by trauma burden, trauma type and sex. We analysed UK Biobank data from 152,863 participants (mean age = 56.4 years; 56.5% female). Lifetime exposure to six non-relational traumatic experiences was assessed. Biological ageing markers included metabolomic age (MileAge) delta, a metabolomic mortality profile score, frailty, leukocyte telomere length and grip strength. Regression models, adjusted for demographic and socioeconomic confounders, estimated associations between trauma and biological ageing markers. We also examined trauma burden, trauma type-specific and sex-specific associations. Non-relational trauma was associated with a metabolite-predicted age exceeding chronological age (MileAge delta; *β* = 0.047, 95% CI 0.032–0.062), elevated metabolomic mortality scores (*β* = 0.102, 95% CI 0.051–0.153) and greater frailty (*β* = 0.298, 95% CI 0.290–0.307), with a graded, approximately linear pattern for frailty (i.e., higher non-relational trauma sum scores were associated with higher frailty scores). All trauma types were associated with greater frailty, with the strongest association for life-threatening illness. There was no evidence of associations with telomere length, and mixed findings for grip strength. Several associations differed by sex, for example overall trauma burden was more strongly associated with greater frailty in females compared to males. Lifetime non-relational trauma was associated with older biological ageing profiles, with the strongest associations with frailty. These findings support the notion that non-relational trauma exposure is associated with long-term health status, underscoring the need for mitigating ageing-related health decline in trauma-exposed populations.

**Supplementary Information:**

The online version contains supplementary material available at https://doi.org/10.1007/s11357-026-02110-4.

## Introduction

Non-relational traumatic experiences, such as serious accidents, war or life-threatening illness, can have profound and lasting effects on both mental and physical health [1–3]. Unlike relational trauma, where the perpetrator is known to the trauma survivor, non-relational trauma either involves an unknown perpetrator (e.g., combat exposure) or no perpetrator (e.g., life-threatening illness). Relational and non-relational trauma have both been linked to increased risks of mental disorders, chronic diseases and morbidity [1–3]. Both have also been linked to an increased risk of premature mortality [4] and to metabolic changes [5].

One proposed mechanism linking trauma and adverse health outcomes is accelerated biological ageing, which refers to the progressive accumulation of molecular and cellular damage and the decline of physiological integrity. Biological ageing can be assessed using various molecular, clinical and functional markers [6–8], including ageing clocks, frailty indices, telomere length and grip strength. Each of these measures provides complementary insights into processes of systemic decline. Metabolomics, in particular, is a powerful tool to capture metabolic perturbations shaped by both genetic and environmental exposures [9, 10]. Frailty indices reflect the accumulation of age-related health deficits and are associated with psychosocial stressors [11]. Similarly, telomere attrition and muscular decline have been linked to chronic stress and traumatic experiences [12, 13]. However, associations between non-relational trauma and multiple dimensions of biological ageing remain comparatively underexplored.

Sex differences in life expectancy, health outcomes and stress reactivity are well-documented. On average, females live longer than males despite reporting higher rates of chronic illness and disability in later life [14]. Females also experience higher rates of certain types of traumas, such as sexual assault, whereas males are more often exposed to physical assault and combat-related trauma [15]. Furthermore, biological ageing processes may differ by sex [16, 17]. These observations highlight the importance of examining biological sex as a potential moderator in the relationship between trauma exposure and biological ageing. To the best of our knowledge, no prior studies have examined whether associations of non-relational trauma with biological ageing differ between males and females.

Although there is growing interest in the biological embedding of trauma, much of the existing research has focused on relational trauma or adversity more broadly or has relied on small samples [18]. Studies also often examined single ageing markers in isolation (e.g., [19, 20]), limiting insights into the broader cumulative impact of trauma. Building on our prior study that identified associations between childhood and adulthood adversity and diverse markers of ageing [21], we sought to investigate associations between non-relational trauma and several biological ageing markers in a large, population-based cohort.

Using data from the UK Biobank, we examined whether lifetime exposure to non-relational traumatic experiences is associated with metabolomic ageing, frailty, telomere length and grip strength. We also tested whether associations varied by the total number (i.e., sum score) of trauma exposures, by each trauma type individually (yes/no) or by sex. We hypothesised that non-relational trauma would be associated with older biological ageing profiles in both sexes, with stronger associations for greater trauma burden.

## Methods

### Study population

The UK Biobank recruited over 500,000 individuals from England, Scotland and Wales. At baseline (2006–2010), participants completed health and sociodemographic questionnaires, underwent physical examinations and provided biological samples. Exposure to adversity and trauma was retrospectively assessed via the online follow-up Mental Health Questionnaire (MHQ) administered between 2016 and 2017 [22]. The median interval between the baseline assessment and completion of the MHQ was 7.57 years (IQR = 1.38).

### Non-relational trauma

Lifetime exposure to non-relational trauma was assessed using a six-item checklist [23]. Items included: “In your life, have you … 1) been in a serious accident that you believed to be life-threatening at the time; 2) been involved in combat or exposed to a war-zone (either in the military or as a civilian); 3) been diagnosed with a life-threatening illness; 4) been attacked, mugged, robbed, or been the victim of a physically violent crime; 5) witnessed a sudden violent death (e.g., murder, suicide, aftermath of an accident); 6) been a victim of a sexual assault, whether by a stranger or someone you knew.” Response options were “Never”, “Yes, but not in the last 12 months” and “Yes, within the last 12 months.” Participants were classified as having experienced non-relational trauma (yes/no) if they responded “Yes, but not in the last 12 months” to at least one item. Those who responded “Yes, within the last 12 months” were excluded from analyses as these events occurred after the biomarker data were collected. A sum score (range: 0–6) was derived to reflect overall trauma burden.

### Metabolomic age (MileAge) delta

Nuclear magnetic resonance (NMR) spectroscopy metabolomic biomarkers were quantified in non-fasting plasma samples using the Nightingale Health platform, which measures 249 biomarkers (168 absolute concentrations and 81 derived ratios) via a high-throughput protocol [24]. Technical variation was removed using the ‘ukbnmr’ R package (algorithm v2) [25]. In a prior study [26], we developed a metabolomic ageing clock using a Cubist rule-based regression model. Individual age predictions were aggregated across the ten test sets of the outer nested cross-validation loop to avoid overfitting. Metabolomic age (MileAge) delta was defined as the difference between metabolite-predicted and chronological age, with positive values indicating an older biological age profile.

### Metabolomic mortality profile score

In a separate study, we developed a metabolomic mortality profile score [27]. Complementing the 249 biomarkers directly provided by Nightingale Health, 76 additional lipid, cholesterol and fatty acid ratios were derived [25], yielding a total of 325 biomarkers. A Least Absolute Shrinkage and Selection Operator (LASSO) Cox proportional hazards model predicting time to mortality was developed in English and Welsh participants (*N* = 234,553). The metabolomic mortality profile score was calculated in an out-of-sample dataset of Scottish participants (*N* = 15,788) as a linear combination of the 54 biomarkers with non-zero coefficients in the LASSO Cox model weighted by their estimated log hazard ratios. We used the subset of participants from Scotland for validation to avoid overfitting and because demographic and health-related characteristics vary across the different regions of the UK [28]. Higher scores indicate an elevated mortality risk.

### Frailty index

A frailty index was derived from 49 health deficits reported via touch-screen questionnaires or during nurse-led interviews, in accordance with the following criteria: indicators of poor health, more common in older individuals, neither rare nor universal, covering multiple areas of functioning and available for ≥ 80% of participants [29]. Variables were dichotomised (deficit absent = zero; deficit present = one) or mapped to a score between zero and one, if ordinal. The sum of deficits was divided by the total number of possible deficits, resulting in frailty index values between zero and one, with higher values indicating greater frailty [30]. Participants missing ≥ 10 of 49 variables were excluded [29].

### Telomere length

Leukocyte telomere length was measured using a quantitative polymerase chain reaction (qPCR) assay that expresses telomere length as the ratio of the telomere repeat copy number (T) relative to a single-copy gene (S) encoding haemoglobin subunit beta [31]. T/S ratio is proportional to average telomere length [32]. Measurements were adjusted for operational and technical parameters (PCR machine, staff member, enzyme batch, primer batch, temperature, humidity, primer batch × PCR machine, primer batch × staff member, A260/A280 ratio of the DNA sample and A260/A280 ratio squared), log_*e*_ transformed and *Z*-standardised.

### Grip strength

Maximal grip strength (kilogram force) was measured using a Jamar J00105 hydraulic hand dynamometer (range: 0–90 kg). We used the maximal value from the participant’s self-reported dominant hand. If handedness data were unavailable, the highest value was used.

### Covariates

Potential confounders included chronological age (in years), sex (male/female), highest educational or professional qualification (four levels, reflecting similar years of education: college/university degree; education to age 18 or above, but not reaching degree level (“A levels”/“AS levels” or equivalent, NVQ/HND/HNC or equivalent, other professional qualifications); education to age 16 qualifications (“GCSEs”/“O levels” or equivalent, “CSEs” or equivalent; no qualifications), gross annual household income (< £18,000, £18,000-£30,999, £31,000-£51,999, £52,000-£100,000 or > £100,000), ethnicity (White, Asian, Black, Chinese, Mixed-race or other) and neighbourhood deprivation (Townsend deprivation index, a small-area level measure of socioeconomic status). For details on the corresponding UK Biobank data fields, see our prior publication [33].

### Exclusion criteria

Participants with missing or “prefer not to answer” response to any non-relational trauma item were excluded from the binary and sum score trauma variables. For item-specific analyses, only those missing data for the relevant item were excluded. For categorical covariates, “do not know’, “prefer not to answer” and missing values were coded as a separate missing category. For analyses of MileAge delta, we applied exclusions as per the original study [26]: women pregnant or uncertain of pregnancy status (due to altered metabolite profiles); participants with discordant genetic and self-reported sex (indicating possible data quality issues); and participants with missing or extreme metabolite values (> 4 × the interquartile range from the median).

### Statistical analyses

All analyses were performed in R (version 4.3.0). Descriptive statistics included means and standard deviations or counts and percentages.

Associations between lifetime non-relational trauma (exposure) and each biological ageing marker (MileAge delta, metabolomic mortality profile score, frailty index, telomere length and grip strength) were estimated using ordinary least squares regression. All ageing markers were scaled (mean = 0, standard deviation = 1) to allow for direct comparisons of association estimates. Telomere length and grip strength were reverse coded prior to analysis. We examined a binary (yes/no) exposure definition and an unweighted sum score. To identify potential associations with trauma burden, we further examined associations with exposure to multiple (at least two) trauma types and an ordinal exposure definition (based on the sum score, with levels 5–6 collapsed due to sparse data). For each ageing marker, we fitted a model adjusted for chronological age and sex (Model 1) and model also adjusted for education, income, ethnicity and neighbourhood deprivation (Model 2). *P*-values were corrected for multiple testing using the Benjamini–Hochberg method, with a two-tailed test and a false discovery rate of 5%. To investigate whether specific types of traumas were associated with specific biological ageing markers, separated models were fitted to estimate associations between each trauma type (exposure) and each ageing marker, using the same modelling strategy. Finally, to explore potential sex differences, we estimated sex-stratified associations and assessed potential sex-by-trauma interactions by adding cross-product terms for both the trauma sum score and each trauma type.

### Sensitivity analysis

We performed a sensitivity analysis excluding the item ‘… been a victim of a sexual assault, whether by a stranger or someone you knew’ from the non-relational trauma sum score to avoid potential overlap with our prior study [21]. We then re-ran all analyses using the modified sum score (range: 0–5), including overall associations, sex-stratified models and models testing the interaction between the trauma sum score and sex.

## Results

**Fig. 1:**
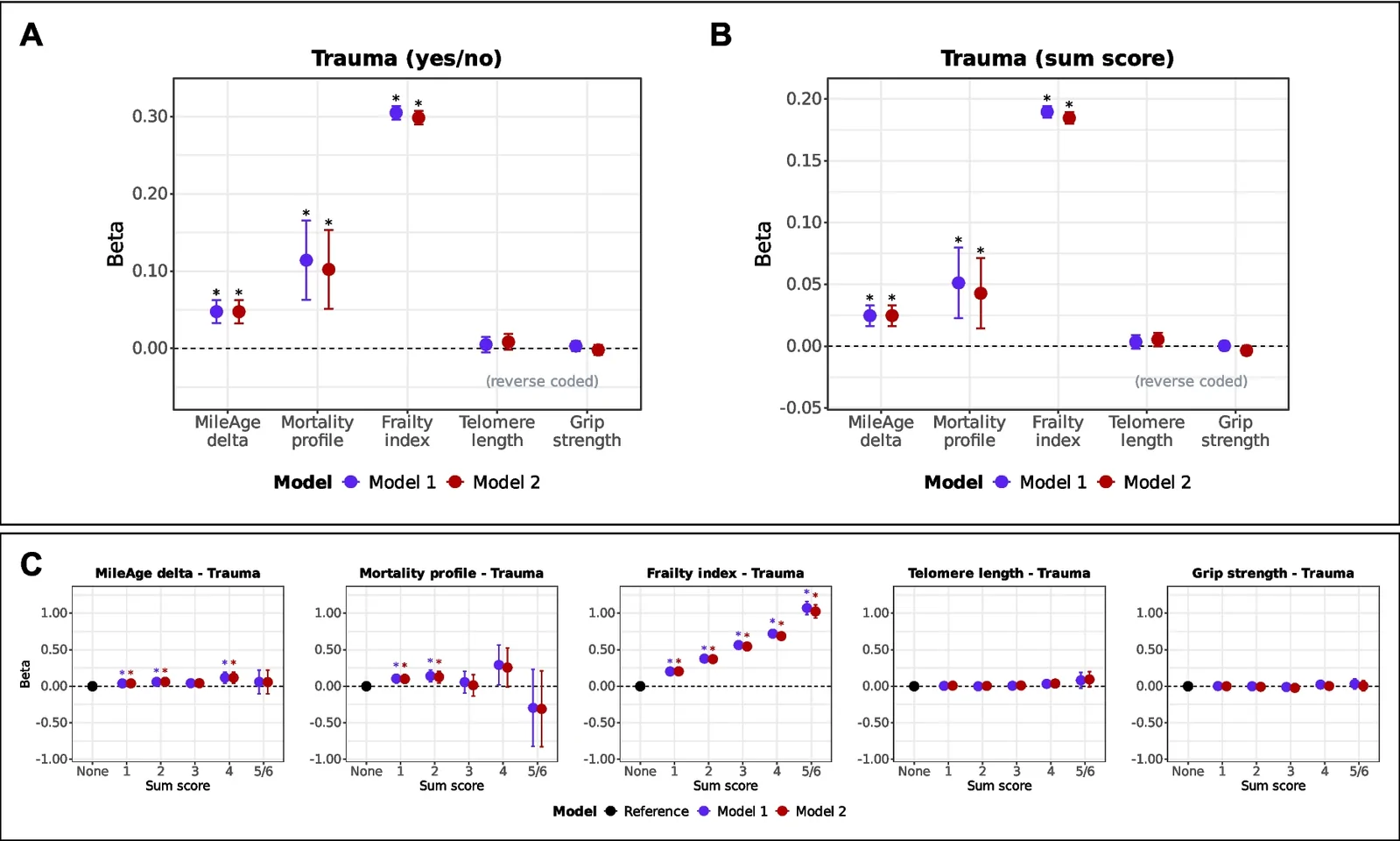
**(A)** Trauma (yes/no) and ageing markers. Associations between lifetime exposure to non-relational trauma and ageing markers (MileAge delta, metabolomic mortality profile, frailty index, telomere length [reverse coded] and grip strength [reverse coded]). Asterisks indicate statistically significant associations, after correcting *p*-values for multiple testing using the Benjamini–Hochberg procedure (across ageing markers and models). **(B)** Trauma (sum scores) and ageing markers. Associations between lifetime exposure to non-relational trauma and ageing markers (MileAge delta, metabolomic mortality profile, frailty index, telomere length [reverse coded] and grip strength [reverse coded]). Asterisks indicate statistically significant associations, after correcting *p*-values for multiple testing using the Benjamini–Hochberg procedure (across ageing markers and models). **(C)** Trauma (sum scores, categorical) and ageing markers. Associations between lifetime exposure to non-relational trauma and ageing markers (MileAge delta, metabolomic mortality profile, frailty index, telomere length [reverse coded] and grip strength [reverse coded]). Asterisks indicate statistically significant associations, after correcting *p*-values for multiple testing using the Benjamini–Hochberg procedure (across exposure levels and models, separately for each ageing marker). **(A-C)** Estimates shown are ordinary least squares regression beta coefficients and 95% confidence intervals. Model 1–adjusted for chronological age and sex; Model 2–adjusted for chronological age, sex, ethnicity, education, income and neighbourhood deprivation. Sample sizes reported in Table S2

Amongst 152,863 participants (mean age = 56.4 years, SD = 7.73; 56.5% female), 77,057 reported lifetime exposure to non-relational trauma (Table S1). Analytical sample sizes for each biological ageing marker are reported in Table S2. Non-relational trauma (yes vs no) was associated with a metabolite-predicted age exceeding chronological age (*β* = 0.047, 95% CI 0.032–0.062, *p* < 0.001), higher metabolomic mortality profile scores (*β* = 0.102, 95% CI 0.051–0.153, *p* < 0.001) and higher frailty index values (*β* = 0.298, 95% CI 0.290–0.307, *p* < 0.001) (Fig. 1A). There was no evidence of associations with telomere length or grip strength (Table 1).

**Table 1:** Associations between non-relational trauma and ageing markers (binary)

	Model 1 (adj. age and sex)				Model 2 (full adjustment)			
	*β*	95% CI		*p*	*β*	95% CI		*p*
Ageing marker								
MileAge delta	0.048	0.033	0.062	< 0.001	0.047	0.032	0.062	< 0.001
Metabolomic profile	0.114	0.063	0.166	< 0.001	0.102	0.051	0.153	< 0.001
Frailty index	0.305	0.296	0.314	< 0.001	0.298	0.290	0.307	< 0.001
Telomere length	0.005	−0.005	0.015	0.411	0.008	−0.002	0.018	0.153
Grip strength	0.003	−0.004	0.010	0.411	−0.002	−0.009	0.004	0.533

CI = confidence interval. Model 1–adjusted for chronological age and sex; Model 2–adjusted for chronological age, sex, ethnicity, highest educational/professional qualification, annual gross household income and Townsend deprivation index. *P*-values shown are corrected for multiple testing using the Benjamini–Hochberg procedure (across ageing markers and models). Sample sizes reported in Table S2

### Associations with trauma burden

**Fig. 2:**
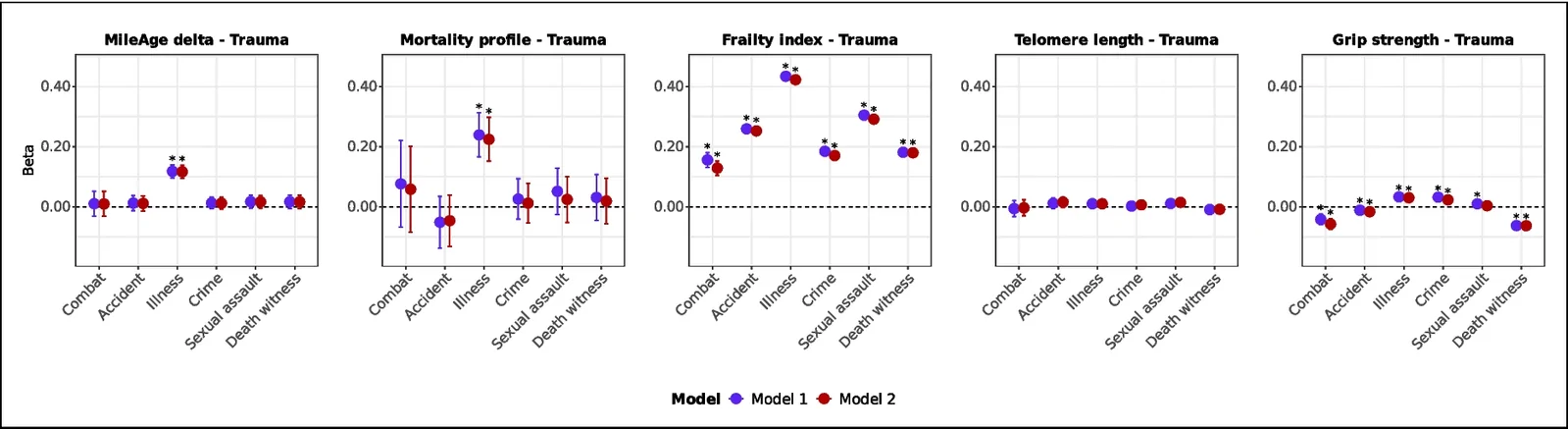
Trauma (item-specific) and ageing markers. Associations between specific trauma items (lifetime exposure to non-relational trauma) and ageing markers (MileAge delta, metabolomic mortality profile, frailty index, telomere length [reverse coded] and grip strength [reverse coded]). Estimates shown are ordinary least squares regression beta coefficients and 95% confidence intervals. Model 1–adjusted for chronological age and sex; Model 2–adjusted for chronological age, sex, ethnicity, education, income and neighbourhood deprivation. Asterisks indicate statistically significant associations, after correcting *p*-values for multiple testing using the Benjamini–Hochberg procedure (across trauma items and models, separately for each ageing marker). Sample sizes reported in Table S2

Exposure to multiple (at least two vs none) non-relational traumas was associated with a MileAge exceeding chronological age, higher metabolomic mortality profile scores and higher frailty index values (Figure S1). Exposure to multiple non-relational traumas was nominally associated with higher grip strength after full adjustment (Table S3). Analyses of the sum scores mirrored the results of the binary exposures (Fig. 1B). There was no evidence of an association with telomere length or grip strength after multiple testing correction (both *p* = 0.073) (Table 2). Sum scores of one, two and four were associated with a metabolite-predicted age exceeding chronological age (Table S4). Sum scores of one and two were also associated with higher metabolomic mortality profile scores. There was a graded, approximately linear association between the number of non-relational trauma exposures and higher frailty index values (ranging from *β* = 0.204, 95% CI 0.194–0.214, *p* < 0.001 to *β* = 1.024, 95% CI 0.933–1.114, *p* < 0.001) (Fig. 2C). There was no evidence that any number of non-relational trauma exposures was associated with telomere length. Finally, sum scores of three were nominally associated with higher grip strength (*β* = −0.022, 95% CI −0.040 to −0.005, *p* = 0.114).

**Table 2:** Associations between non-relational trauma and ageing markers (sum score)

	Model 1 (adj. age and sex)				Model 2 (full adjustment)			
	*β*	95% CI		*p*	*β*	95% CI		*p*
Ageing marker								
MileAge delta	0.025	0.016	0.033	< 0.001	0.025	0.016	0.033	< 0.001
Metabolomic profile	0.051	0.023	0.080	< 0.001	0.043	0.014	0.071	0.005
Frailty index	0.190	0.185	0.194	< 0.001	0.185	0.180	0.189	< 0.001
Telomere length	0.004	−0.002	0.009	0.238	0.005	0.000	0.011	0.073
Grip strength	0.000	−0.003	0.004	0.842	−0.003	−0.007	0.000	0.073

CI = confidence interval. Model 1–adjusted for chronological age and sex; Model 2–adjusted for chronological age, sex, ethnicity, highest educational/professional qualification, annual gross household income and Townsend deprivation index. *P*-values shown are corrected for multiple testing using the Benjamini–Hochberg procedure (across ageing markers and models). Sample sizes reported in Table S2

### Specific types of non-relational trauma

Diagnosis of a life-threatening illness was the only non-relational trauma associated with a MileAge exceeding chronological age (*β* = 0.117, 95% CI 0.095–0.138, *p* < 0.001) and higher metabolomic mortality profile scores (*β* = 0.224, 95% CI 0.151–0.297, *p* < 0.001) (Table S5). All non-relational trauma exposures were associated with higher frailty index values, with the strongest association observed for life-threatening illness (*β* = 0.422, 95% CI 0.410–0.435, *p* < 0.001) (Fig. 2). No associations between non-relational trauma and telomere length were statistically significant after multiple testing correction (*p* ≥ 0.371). We identified mixed associations between non-relational trauma and grip strength. Life-threatening illness and having been a victim of crime were both associated with weaker grip strength. However, combat or warzone exposure, serious accidents or witnessing a sudden violent death were all associated with higher grip strength.

### Sex differences

Amongst 152,863 participants, 46.3% of females (39,950 out of 86,311) and 55.8% of males (37,107 out of 66,552) reported lifetime exposure to non-relational trauma (Table S6). Males had higher trauma sum scores than females (Table S7). Combat exposure was more common amongst males (6.1%) than females (1.5%), whereas sexual assault was more prevalent amongst females (20.6%) than males (7.6%). Analytical sample sizes are shown in Table S7.

**Fig. 3:**
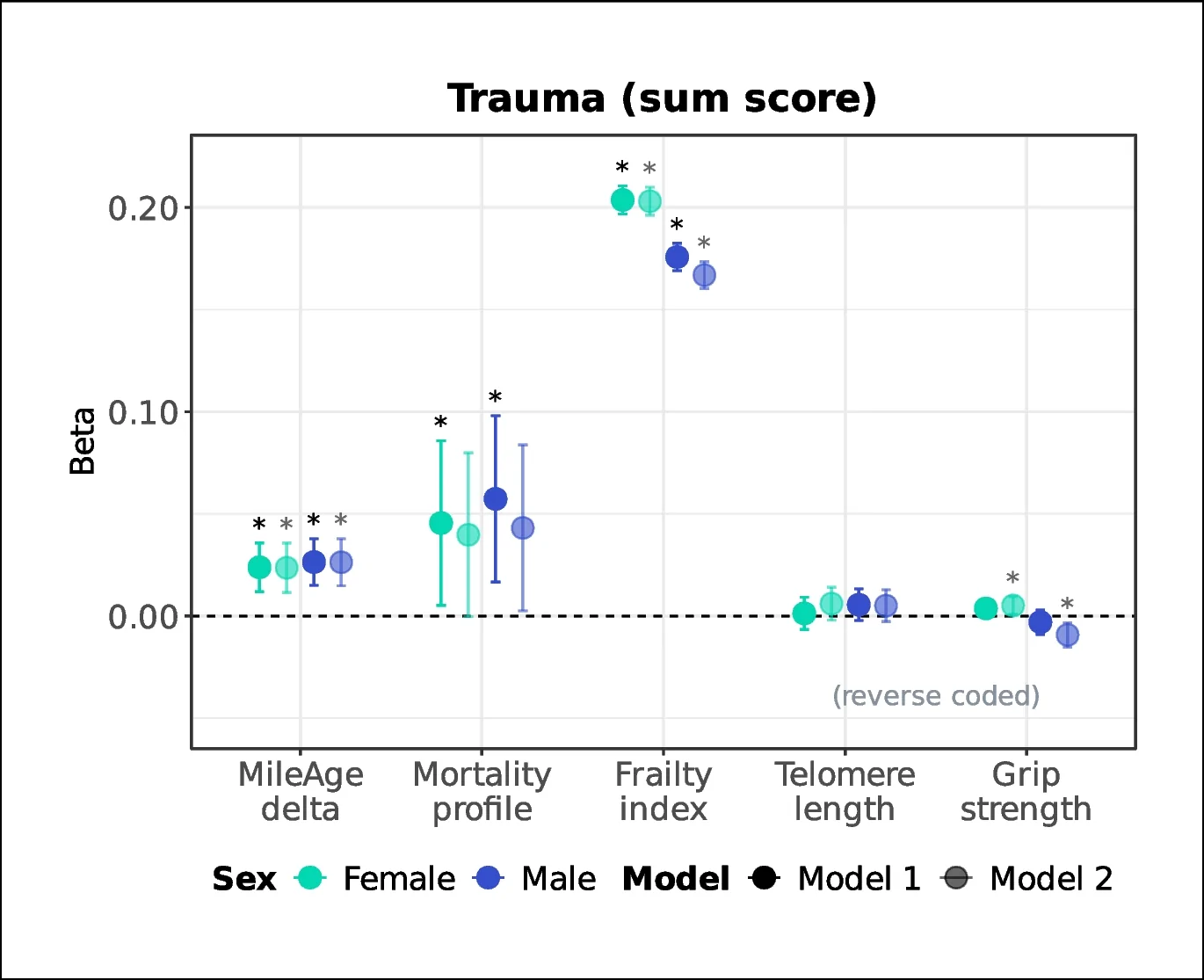
Sex-stratified associations between lifetime exposure to non-relational trauma (sum scores) and ageing markers (MileAge delta, metabolomic mortality profile, frailty index, telomere length [reverse coded] and grip strength [reverse coded]). Estimates shown are ordinary least squares regression beta coefficients and 95% confidence intervals. Model 1–adjusted for chronological age; Model 2–adjusted for chronological age, ethnicity, highest educational/professional qualification, annual gross household income and neighbourhood deprivation. Asterisks indicate statistically significant associations, after correcting *p*-values for multiple testing using the Benjamini–Hochberg procedure (across ageing markers and models, separately for each sex). Sample sizes reported in Table S7

In sex-stratified analyses, associations between non-relational trauma and metabolomic ageing were comparable in males and females (Fig. 3; Table S8). Associations with frailty were stronger in females (*β* = 0.203, 95% CI 0.196–0.210, *p* < 0.001) than in males (*β* = 0.167, 95% CI 0.160–0.173, *p* < 0.001), with a statistically significant interaction (*β*_interaction_ = −0.035, 95% CI −0.044 to −0.025, *p* < 0.001) (Table S9). After full adjustment, non-relational trauma (sum score) was associated with lower grip strength in females (*β* = 0.005, 95% CI 0.001–0.009, *p* = 0.037) and with higher grip strength in males (*β* = −0.009, 95% CI −0.015 to −0.003, *p* = 0.005). However, the interaction was not statistically significant (*β*_interaction_ = −0.007, 95% CI −0.014 to 0.000, *p* = 0.146).

**Fig. 4:**
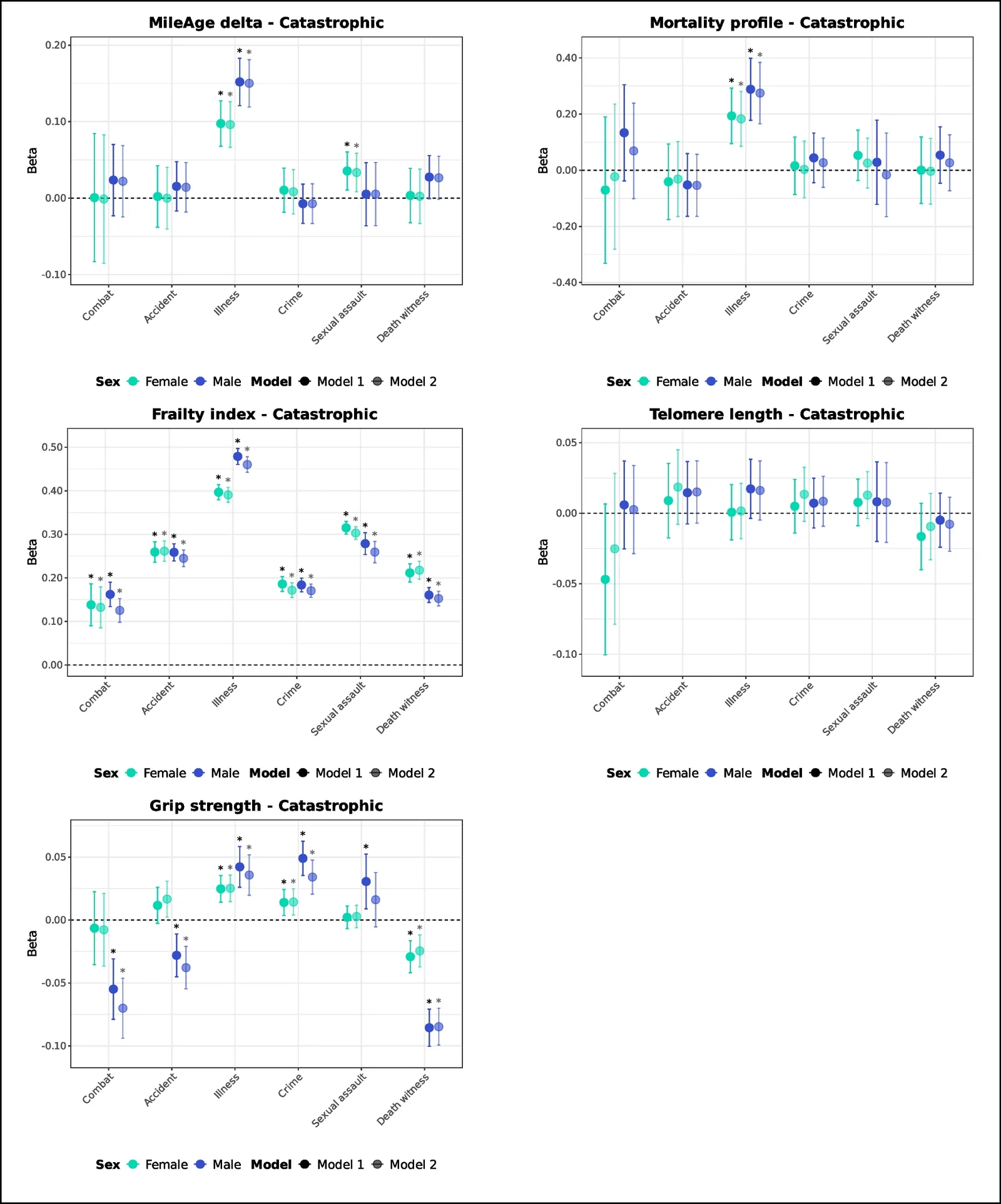
Sex-stratified associations between specific lifetime exposure to non-relational trauma items and ageing markers (MileAge delta, metabolomic mortality profile, frailty index, telomere length [reverse coded] and grip strength [reverse coded]). Estimates shown are ordinary least squares regression beta coefficients and 95% confidence intervals. Model 1–adjusted for chronological age; Model 2–adjusted for chronological age, ethnicity, highest educational/professional qualification, annual gross household income and neighbourhood deprivation. Asterisks indicate statistically significant associations, after correcting *p*-values for multiple testing using the Benjamini–Hochberg procedure (across exposures, ageing markers and models, separately for each sex). Sample sizes reported in Table S7

Life-threatening illness was numerically more strongly associated with MileAge delta in males (*β* = 0.150, 95% CI 0.119–0.181, *p* < 0.001) than in females (*β* = 0.096, 95% CI 0.066–0.126, *p* < 0.001) (Fig. 4; Table S10), though the interaction was not statistically significant (*p*_interaction_ > 0.999) (Table S11). Sexual assault was associated with a metabolite-predicted age exceeding chronological age only in females (*β* = 0.034, 95% CI 0.008–0.059, *p* = 0.022). The metabolomic mortality profile score was also numerically more strongly associated with life-threatening illness in males (*β* = 0.275, 95% CI 0.165–0.384, *p* < 0.001) than in females (*β* = 0.183, 95% CI 0.085–0.281, *p* < 0.001), although the interaction test was again not statistically significant (*p*_interaction_ = 0.305). Life-threatening illness was more strongly associated with frailty in males (*β* = 0.460, 95% CI 0.442–0.478, *p* < 0.001) than in females (*β* = 0.390, 95% CI 0.374–0.407, *p* < 0.001), with a statistically significant interaction (*β*_interaction_ = 0.068, 95% CI 0.044–0.092, *p* < 0.001). Sexual assault and witnessing a sudden violent death were more strongly associated with frailty in females (*β* = 0.303, 95% CI 0.289–0.318, *p* < 0.001 and *β* = 0.218, 95% CI 0.197–0.238, *p* < 0.001, respectively) than males (*β* = 0.259, 95% CI 0.235–0.284, *p* < 0.001 and *β* = 0.153, 95% CI 0.136–0.169, *p* < 0.001, respectively), with statistically significant interactions (*β*_interaction_ = −0.040, 95% CI −0.068 to −0.011, *p* = 0.034; *β*_interaction_ = −0.065, 95% CI −0.091 to −0.038, *p* < 0.001). Combat or war-zone exposure (*β* = −0.070, 95% CI −0.094 to −0.046, *p* < 0.001) and serious accidents (*β* = −0.038, 95% CI −0.055 to −0.021, *p* < 0.001) were associated with higher grip strength only in males. A statistically significant interaction was detected for serious accidents (*β*_interaction_ = −0.043, 95% CI −0.065 to −0.021, *p* < 0.001). Witnessing a sudden violent death was associated with higher grip strength in both sexes but the association was stronger in males (*β*_interaction_ = −0.058, 95% CI −0.078 to −0.039, *p* < 0.001). Finally, associations between life-threatening illness or physical violence and lower grip strength were stronger in males, with a statistically significant interaction for physical violence (*β*_interaction_ = 0.033, 95% CI 0.017–0.050, *p* < 0.001).

### Sensitivity analysis excluding sexual assault

After excluding sexual assault from the sum score, results were largely consistent with the main findings. After full adjustment, non-relational trauma was associated with higher grip strength (*β* = −0.005, 95% CI −0.009 to −0.001, *p* = 0.027) (Figure S2; Table S12). In sex-stratified analyses, trauma was associated with elevated metabolomic mortality profile scores in males after full adjustment (*β* = 0.053, 95% CI 0.010–0.096, *p* = 0.023), whereas in females this association was not statistically significant in either model (*p* ≤ 0.083) (Figure S3; Table S13). Finally, there was evidence of a statistically significant interaction between trauma and sex for grip strength (*β*_interaction_ = −0.012, 95% CI −0.020 to −0.004, *p* = 0.013) (Table S14).

## Discussion

This study demonstrates that exposure to non-relational trauma is associated with older biological ageing profiles across multiple ageing markers, including metabolomic ageing, frailty and grip strength. The strongest and most consistent association across all trauma types was observed for frailty, with a graded, approximately linear pattern. The use of a large, population-based sample from the UK Biobank allowed for greater statistical power to detect even moderate effect sizes compared to prior research, which has often relied on smaller samples (e.g., [34, 35]). By examining multiple markers of biological ageing, and by distinguishing between trauma types and cumulative exposure levels, our findings provide more comprehensive insights into biological ageing as a potential mechanism underlying the long-term health consequences of trauma exposure.

Non-relational trauma was associated with a metabolite-predicted age exceeding chronological age. Metabolomic ageing clocks capture systemic metabolic dysregulation and cumulative biological stress [10]. The observation that exposure to non-relational trauma was associated with an older metabolomic age suggests that such experiences may trigger or accelerate metabolic dysfunction, potentially through sustained activation of the hypothalamic–pituitary–adrenal axis, increased oxidative stress or inflammation [36, 37]. This aligns with prior research linking trauma to dysregulation in metabolic and inflammatory pathways and underscores the potential of metabolomics as a tool to capture trauma-related changes in biological ageing [5, 38]. Similarly, the metabolomic mortality profile score, a composite index trained on survival data, was elevated amongst trauma-exposed individuals. This supports the notion that trauma can have enduring consequences for health. This finding aligns with epidemiological studies identifying an increased mortality risk amongst trauma survivors and further substantiates the biological embedding of traumatic experiences [39]. For both measures, life-threatening illness was the only non-relational trauma associated with advanced metabolomic ageing. Importantly, life-threatening illness per se should not be conceptualised as trauma, as associations with metabolomic ageing are likely driven, at least in part, by disease-related metabolic dysregulation rather than trauma-related biological embedding. Rather, only those illness-related experiences that involve sudden threat to life, severe functional loss or intense psychological distress may meet criteria for traumatic exposure. Future research should examine whether the perceived life‑threatening nature of illness and its psychological impact contribute to biological ageing beyond the direct physiological effects of disease.

In contrast, associations between trauma and frailty were broad, consistent and graded, supporting frailty as the most robust measure to capture the possible impact of trauma on biological ageing. Frailty, as operationalised in this study, summarises the accumulation of age-related health deficits and functional impairments. Its sensitivity to trauma exposure highlights the broader impact that traumatic experiences may have beyond psychological outcomes such as post-traumatic stress. Our results mirror recent findings that link social stress and disadvantage with frailty in mid-to-late adulthood, suggesting that trauma may accelerate biological ageing processes via compounded physiological stress across systems [40]. Every trauma type was associated with greater frailty, with the strongest association observed for life-threatening illness. This likely reflects the cumulative impact of somatic disease burden on frailty, alongside potential psychological stress responses, rather than trauma exposure alone.

There was no evidence that telomere length was associated with lifetime exposure to non-relational trauma. This finding is inconsistent with most studies in the field, which found greater telomere attrition in populations exposed to trauma or adversity [19, 41, 42]. It is also inconsistent with our previous finding of an association between adversity experienced in childhood and shorter telomeres [21]. This inconsistency with prior studies may reflect differences in sample characteristics, trauma definition and timing of exposure. For example, in our prior study [21] we examined adversity and relational trauma more broadly, whereas in the current study we focused only on non-relational trauma, which may differentially impact biological ageing.

There was also limited evidence that grip strength was associated with overall lifetime exposure to non-relational trauma. Grip strength is a strong predictor of disability and mortality and reflects the integrity of multiple systems, including neuromuscular, cardiovascular and inflammatory pathways [43–45]. The findings reported here are largely inconsistent with our previous study generally indicating lower grip strength associated with adversity and relational trauma experienced in childhood and adulthood [21]. This may be due to differences in the type and severity of exposure. Again, the present study focused on non-relational trauma only, which encompassed experiences such as military combat that may be more common among physically stronger individuals. Indeed, when examining specific types of non-relational trauma, we found that combat exposure, serious accidents or witnessing violent deaths were associated with higher grip strength, whereas life-threatening illness or being a victim of a crime were associated with lower grip strength.

Importantly, a graded, approximately linear relationship was evident for frailty and, to a lesser extent, for the metabolomic clock and mortality profile score, with exposure to multiple (vs single and vs no) traumatic experiences corresponding to older biological ageing profiles. This cumulative effect is consistent with allostatic load theory, which posits that repeated or sustained stress results in wear and tear across physiological systems [40]. The finding that individuals exposed to multiple types of non-relational trauma had older biological profiles than individuals exposed to one trauma type (and even more so than individuals not exposed to any lifetime trauma) provides empirical support for the idea of a dose-dependent impact of trauma on biological ageing.

We also identified differences in how non-relational trauma relates to biological ageing across males and females. While non-relational trauma was associated with greater frailty in both sexes, the association was stronger in females, particularly for females reporting sexual assault and/or witnessing a sudden violent death. In contrast, associations between life-threatening illness and the metabolomic clock or mortality profile score were more pronounced in males. These findings suggest that sex may moderate the biological embedding of trauma, underscoring the importance of sex-sensitive approaches in trauma research and intervention. Grip strength also showed sex-specific patterns: non-relational trauma was associated with lower grip strength in females but higher strength in males, possibly reflecting differential exposure to certain trauma types. Future research should address the possibility that associations between combat, accident and sudden violent death exposure and higher grip strength in males reflect selection effects rather than biological embedding.

Our findings contribute to a growing literature on the biological embedding of trauma. While previous studies have demonstrated that early-life adversity can alter stress physiology, immune function and cellular ageing [46, 47], fewer studies have examined adulthood trauma, particularly non-relational events [2]. Moreover, prior studies have typically relied on single ageing biomarkers in small, clinical samples, limiting generalisability and the ability to capture system-wide effects [18]. This study addresses these gaps using a large, community-based cohort and a multidimensional set of ageing markers, thereby offering a more comprehensive picture of the links between trauma and biological ageing. Importantly, future research should investigate whether trauma survivors who develop lasting mental health conditions (e.g., post-traumatic stress disorders or major depression) exhibit poorer biological outcomes compared to trauma survivors without such conditions.

These findings also have public health and clinical implications. They suggest that non-relational trauma is not only a psychological burden but also associated with biological ageing processes, which, when dysregulated, are linked to premature mortality [26]. Early identification and support for individuals with trauma exposure may help mitigate some of these long-term biological consequences. Interventions aimed at reducing chronic stress, promoting physical activity, enhancing social support and addressing metabolic dysfunction may be particularly beneficial for trauma-exposed populations [48]. Perhaps the need for prevention and intervention might be particularly high for those who develop mental disorders in the aftermath of trauma such as post-traumatic stress disorder and fortunately effective interventions exist [49]. Yet, the question of whether and how trauma-related mental illnesses relate to the link between trauma and adverse physiological outcomes remains understudied for the time being.

Several limitations should be acknowledged. First, trauma exposure was retrospectively self-reported, which may introduce recall bias or underreporting. Yet, retrospective compared to prospective accounts of trauma exposure have been shown to be the clinically more useful predictors of adverse (mental) health outcomes [50]. Second, the categorisation of trauma based on lifetime recall rather than timing (e.g., in childhood and/or adulthood [21]), may obscure important nuances in the exposure–response relationship. Future research should assess non-relational trauma experienced in childhood and/or adulthood to test whether associations with biological ageing markers vary by timing of exposure. Similarly, severity and frequency of non-relational trauma were not assessed in the UK Biobank and should be investigated in future studies. In our analysis of exposure to multiple traumas, we derived the categorisation based on the number of different trauma types. In future studies, the number of events per trauma type would provide a more fine-grained approach to assessing trauma burden. Third, the cross-sectional study design limits causal inference. Although the observed associations are biologically plausible and align with prior longitudinal research [51, 52], we cannot conclude that trauma causes differences in biological ageing markers. For example, it is possible that individuals with poor health and perturbed biological ageing markers are more likely to perceive an illness as life-threatening. Fourth, while we adjusted for multiple demographic and socioeconomic confounders, residual confounding is possible. For example, genetic predispositions or behavioural factors could influence both trauma exposure and biological ageing. Importantly, mental disorders are associated with various biological ageing markers [53, 54] and may serve as a mediator linking trauma exposure to variations in biological ageing. Formally testing for mediation was beyond the scope of the present study. Future studies should test the extent to which mental disorders mediate associations between trauma exposure and biological ageing. For the trauma type-specific analyses, we note further complexities that cannot be disentangled by the current study design and data availability: life-threatening illness may conflate the somatic consequences of illness exposure with its psychological impact; serious accidents may correlate with occupational factors and/or risk-taking behaviours (such as substance use), the latter of which is also linked to older biological ageing [53, 55]; and positive associations between grip strength and warzone or combat exposure in males may reflect selection into military service, itself linked to physical fitness and other confounding factors such as socioeconomic status. Fifth, the UK Biobank is not fully representative of the general UK population; participants are generally healthier, wealthier and more educated, which may bias association estimates. Finally, the analysis of the metabolomic mortality profile score was restricted to the subset of participants recruited at the two assessment centres in Scotland. This was due to the original study design [27], to avoid overfitting and to validate the score in a sample that differed in terms of socio-economic and health characteristics from the training sample. In the future, out-of-sample scores could be calculated in the full UK Biobank using nested cross-validation, as we have done for the MileAge clock [26].

In conclusion, this study provides robust evidence that non-relational trauma is associated with older biological ageing profiles across multiple domains, most clearly reflected in greater frailty across all trauma types in a graded, approximately linear fashion. These findings underscore the need to recognize trauma as a significant contributor to long-term physical health and biological ageing, warranting further investigation and exploring preventative and intervention strategies. Future research should focus on longitudinal designs, mechanistic pathways and modifiable resilience factors that could buffer the biological impact of trauma.

## Supplementary Information

Below is the link to the electronic supplementary material.

Supplementary file1 (DOCX 269 KB)

## Data Availability

The data used are available to all *bona fide* researchers for health-related research that is in the public interest, subject to an application process and approval criteria. Study materials are publicly available online at http://www.ukbiobank.ac.uk.
